# When directed evolution met ancestral enzyme resurrection

**DOI:** 10.1111/1751-7915.12452

**Published:** 2016-11-11

**Authors:** Miguel Alcalde

**Affiliations:** ^1^ Department of BiocatalysisInstitute of CatalysisCSICCantoblanco28049MadridSpain

## Abstract

The directed evolution of ancestral ‐resurrected‐ enzymes can give a new twist in protein engineering approaches towards more versatile and robust biocatalysts.

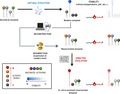

On the twenty‐fifth anniversary of the invention of directed molecular evolution, few can doubt the power of this revolutionary method to engineer useful biomolecules at the service of mankind. By compressing Darwin's concept of selection through ‘*survival of the fittest*’ into a test tube, it has become possible to design enzymes *a la carte*, enzymes that can be used in pharmaceutical applications or to produce agrochemicals, biofuels and daily products. Through iterative rounds of random mutation, recombination and screening, evolved and improved enzymes have been incorporated into many aspects of our lives, gradually replacing noxious chemicals and high energy demanding steps of industrial production pipelines in the drive towards green chemistry. Thanks to the exponential growth in protein and genome databases, and the blossoming array of predictive computational methods, we are now for the first time able to travel beyond the frontiers of nature through the directed evolution of enzymes (including *de novo* enzymes), metabolic pathways, genetic circuits or whole cells to be used in a wide range of biotechnological applications. As such, we can tailor enzymes to perform non‐natural chemistry or to adapt them to non‐natural environments, as well as other practical cases in bioremediation, biomedicine or bionanomaterials, to name but a few (Molina‐Espeja *et al*., 2016).

By contrast, enzyme reconstruction and resurrection has recently emerged as an approach to rapidly obtain potential biocatalysts. Indeed, using phylogenetic analysis and ancestral inference algorithms, versions of ancient enzymes are being rapidly recreated. Among them, we can find thioredoxins, alcohol dehydrogenases, beta‐lactamases or rubiscos (mostly 1–3 billion years old Precambric origin), whose stability and/or wider substrate range may make them more suitable workhorses for further directed evolution towards more challenging applications (Perez‐Jimenez *et al*., 2011; Carrigan *et al*., 2012; Risso *et al*., 2013; Shih *et al*., 2016). The remarkable ancestral properties of these enzymes are thought to be linked to the unpleasant environment that organisms faced on the earth during the Precambrian age (e.g. high temperatures (Akanamura *et al*., 2013)) and to the assumption that primitive cells relied on only a small array of enzymes because their physiology made it impossible for them to produce specialist enzymes for each metabolic task. Thus, it is thought that ancient enzymes were both robust and promiscuous, working like a ‘*Swiss army knife*’ to fulfill a plethora of activities. Similarly, they represent good blueprints suitable to adaptation, both promoting the survival of the cell while eventually becoming more specialized over the course of natural evolution.

Whether or not the recent resurrected products obtained in the laboratory are reliable approximations to the true extinct enzyme phenotypes is still open to debate. Indeed, there are two main currents of opinion regarding this controversial issue: (i) those who believe that the properties of the resurrected enzymes are mostly due to the accumulation of consensus/ancestor mutations, such that similar properties could be achieved by introducing such mutations into their modern counterparts (e.g. by classical consensus mutagenesis); and (ii) those who believe that the properties of the resurrected enzymes are not exclusively dependent on the set of consensus/ancestor mutations but rather, they reflect the broad differences in protein sequence with respect to the extant enzymes (sometimes sharing as little as ~50% sequence identity despite sharing overall structural motives and similar folding) (Cole and Gaucher, 2011; Risso *et al*., 2014). While this debate remains open, *paleoenzymologists* are harnessing resurrected enzymes to decipher some of the principles of natural protein evolution, just as directed evolution has sometimes done with modern enzymes (Bloom and Arnold, 2009).

From a strictly biotechnology viewpoint, the fledgling association of ancestral enzyme resurrection and directed evolution is very timely, especially given that the combination of both methods may yield stronger and more versatile biocatalysts (Arnaud, 2013; Alcalde, 2015). For example, the directed evolution of resurrected enzymes could help rescue latent/promiscuous activities lost during natural evolution but that could be used today for biotechnological purposes, Fig. 1. Moreover, given that resurrected enzymes are very stable (in terms of tolerance to extreme pH or high temperature – sometimes with a *T*_m_ >30°C higher than their modern counterparts) and that beneficial mutations are typically destabilizing, these ancestral proteins represent wonderful virgin moulds for more aggressive protein engineering approaches that unfortunately most modern enzymes cannot withstand. Indeed, we recently found these enzymes to be more tolerant to high mutational loads in directed evolution campaigns, such as when evolving resurrected CO_2_‐fixing enzymes or different types of ancestral ligninases (unpublished data).

**Figure 1:**
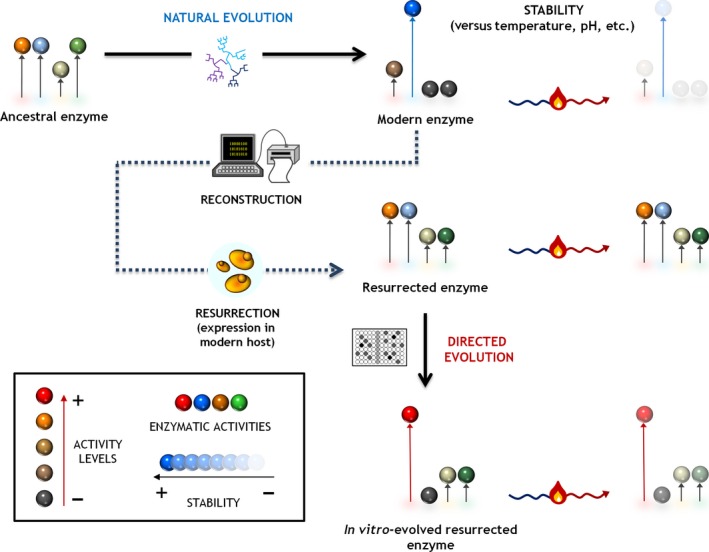
Directed evolution of ancestral enzymes. From a modern enzyme scaffold, a plausiable approximation to the ancestral node is predicted through bioinformatic computation (protein sequence reconstruction). The sequence of the ancestral node is then synthesized and functional expressed (resurrected) in a suitable host (e.g. bacteria or yeast). Thereafter, the resurrected enzyme is subjected to directed evolution towards latent activities those are hardly shown by the modern counterpart. The strong stability of the resurrected node can help alleviate the detrimental effect of using high mutational loads during the directed evolution campaign.

The growth and impact of directed evolution in all areas of biotechnology is becoming overwhelming, such that a unique opportunity has become reality before our very eyes, enabling us to design powerful enzymes that will gradually replace chemical catalysts. As Prof. Frances H. Arnold from Caltech, pioneer in the field of directed evolution, recently said during the Millenium Technology Prize Ceremony ‘Nature is this innovation machine. We have this capability to re‐write the code of life. It doesn't have to be a new opportunity for exploitation…it should be an opportunity for exploration and for coming up with new solutions to the problems that we have’. Most researchers in the field fully agree with this statement and so, it is reasonable to think that not only extant enzymes but also their resurrected counterparts could be evolved on the laboratory bench in the near future, allowing us to travel back and forth along the evolutionary timeline. Thus, we will soon be joyously reaping the consequences of the meeting between directed evolution and ancestral enzyme resurrection.

## References

[mbt212452-bib-0001] Akanamura, S. , Nakajima, Y. , Yokobori, S. , Kimura, M. , Nemoto, N. , Mase, T. , *et al* (2013) Experimental evidence for the thermophilicity of ancestral life. Proc Natl Acad Sci USA 110: 11067–11072. 23776221 10.1073/pnas.1308215110 PMC3703998

[mbt212452-bib-0002] Alcalde, M. (2015) Engineering the ligninolytic consortium. Trends Biotechnol 33: 155–162. 25600621 10.1016/j.tibtech.2014.12.007

[mbt212452-bib-0003] Arnaud, C.H. (2013) Bringing ancient proteins to life. C&EN 8: 38–39.

[mbt212452-bib-0004] Bloom, J.D. , and Arnold, F.H. (2009) In the light of directed evolution: pathways of adaptive protein evolution. Proc Natl Acad Sci USA 106: 9995–10000. 19528653 10.1073/pnas.0901522106 PMC2702793

[mbt212452-bib-0005] Carrigan, M.A. , Uryasev, O. , Davis, R.P. , Zhai, L. , Hurley, T.D. , and Benner, S.A. (2012) The natural history of classs I primate alcohol dehydrogenases includes gene duplication, gene loss, and gene conversion. PLoS ONE 7: e41175. 22859968 10.1371/journal.pone.0041175 PMC3409193

[mbt212452-bib-0006] Cole, M.F. , and Gaucher, E.A. (2011) Utilizing natural diversity to evolve protein function: applications towards thermostability. Curr Opin Chem Biol 15: 399–406. 21470898 10.1016/j.cbpa.2011.03.005 PMC3173975

[mbt212452-bib-0007] Molina‐Espeja, P. , Viña‐Gonzalez, J. , Gomez, B.J. , Martin‐Diaz, J. , Garcia‐Ruiz, E. , and Alcalde, M. (2016) Beyond the outer limits of nature by directed evolution. Biotechnol Adv 34: 754–767. 27064127 10.1016/j.biotechadv.2016.03.008

[mbt212452-bib-0008] Perez‐Jimenez, R. , Ingles‐Prieto, A. , Zhao, Z.‐M. , Sanchez‐Romero, I. , Alegre‐Cebollada, J. , Kosuri, P. , *et al* (2011) Single‐molecule paleoenzymology probes the chemistry of resurrected enzymes. Nat Struct Mol Biol 18: 592–597. 21460845 10.1038/nsmb.2020 PMC3087858

[mbt212452-bib-0009] Risso, V.A. , Gavira, J.A. , Mejia‐Carmona, D.F. , Gaucher, E.A. , and Sanchez‐Ruiz, J.M. (2013) Hyperstability and substrate promiscuity in laboratory resurrections of Precambrian β‐lactamases. J Am Chem Soc 135: 2899–2902. 23394108 10.1021/ja311630a

[mbt212452-bib-0010] Risso, V.A. , Gavira, J.A. , Gaucher, E.A. , and Sanchez‐Ruiz, J.M. (2014) Phenotypic comparison of consensus variants versus laboratory resurrections of Precambrian proteins. Proteins 82: 887–896. 24710963 10.1002/prot.24575

[mbt212452-bib-0011] Shih, P.M. , Occhialini, A. , Cameron, J.C. , Andralojc, P.J. , Parry, M.A.J. , and Kerfeld, C.A. (2016) Biochemical characterization of predicted Precambrian RuBisCO. Nat. Comm. 7: 10382. 10.1038/ncomms10382 PMC4735906 26790750

